# Autoimmune Global Amnesia as Manifestation of AMPAR Encephalitis and Neuropathologic Findings

**DOI:** 10.1212/NXI.0000000000001019

**Published:** 2021-05-20

**Authors:** Gerda Ricken, Tobias Zrzavy, Stefan Macher, Patrick Altmann, Johannes Troger, Kim Kristin Falk, Andreas Kiefer, Andreas Fichtenbaum, Goran Mitulovic, Helmut Kubista, Klaus-Peter Wandinger, Paulus Rommer, Thorsten Bartsch, Thomas Berger, Jörg Weber, Frank Leypoldt, Romana Höftberger

**Affiliations:** From the Division of Neuropathology and Neurochemistry (G.R., A.F., R.H.), Department of Neurology, Medical University of Vienna, Austria; Department of Neurology (T.Z., S.M., P.A., P.R., T. Berger), Medical University of Vienna, Austria; Department of Neurology (J.T., J.W.), Klinikum Klagenfurt, Austria; Institute of Clinical Chemistry (K.K.F., K.-P.W., F.L.), University Hospital Schleswig-Holstein, Kiel/Lübeck, Germany; Institute of Pathology (A.K.), Klinikum Klagenfurt, Austria; Clinical Department of Laboratory Medicine (A.F., G.M.), Proteomics Core Facility, Medical University Vienna, Austria; Center of Physiology and Pharmacology (H.K.), Department of Neurophysiology and Neuropharmacology, Medical University of Vienna, Austria; and Department of Neurology (T. Bartsch, F.L.), University Hospital Schleswig-Holstein, Kiel, Germany.

## Abstract

**Objective:**

To report an unusual clinical phenotype of alpha-amino-3-hydroxy-5-methyl-4-isoxazolepropionic acid receptor (AMPAR) encephalitis and describe associated neuropathologic findings.

**Methods:**

We retrospectively investigated 3 AMPAR encephalitis patients with autoimmune global hippocampal amnesia using comprehensive cognitive and neuropsychologic assessment, antibody testing by in-house tissue-based and cell-based assays, and neuropathologic analysis of brain autopsy tissue including histology and immunohistochemistry.

**Results:**

Three patients presented with acute-to-subacute global amnesia without affection of cognitive performance, attention, concentration, or verbal function. None of the patients had epileptic seizures, change of behavior, personality changes, or psychiatric symptoms. The MRI was normal in 1 patient and showed increased fluid-attenuated inversion recovery/T2 signal in the hippocampus in the other 2 patients. Two patients showed complete remission after immunotherapy. The one patient who did not improve had an underlying adenocarcinoma of the lung and died 3.5 months after disease onset because of tumor progression. Neuropathologic analysis of the brain autopsy revealed unilateral hippocampal sclerosis accompanied by mild inflammatory infiltrates, predominantly composed of T lymphocytes, and decrease of AMPAR immunoreactivity.

**Conclusion:**

AMPAR antibodies usually associate with limbic encephalitis but may also present with immune responsive, acute-to-subacute, isolated hippocampal dysfunction without overt inflammatory CSF or MRI changes.

Autoantibodies to the GluA1 and/or GluA2 subunit of the alpha-amino-3-hydroxy-5-methyl-4-isoxazolepropionic acid receptor (AMPAR) were originally identified in patients with limbic encephalitis with prominent behavioral and psychiatric changes and epileptic seizures.^1^ Up to 64% have an underlying malignancy, most commonly thymoma, lung, or breast cancer.^2-4^ Subsequently, published studies expanded the spectrum of neurologic deficits to focal weakness, involuntary movements, autonomic dysfunction, upper motor neuron signs, apraxia, aphasia, sensory symptoms, or ataxia as additional symptoms apart from the limbic system.^2,4^ Recently, we identified AMPAR antibodies (abs) in the CSF of a 31-year-old patient, who presented with a clinical picture reminiscent of transient global amnesia without associated neurologic signs or overt inflammatory CSF or MRI changes and who readily responded to immunotherapy. This observation and 2 subsequent patients with similar phenotypes that came to our attention triggered this study. Our aim was to describe this unusual and possibly overlooked clinical presentation of AMPAR encephalitis, we tentatively dubbed autoimmune global hippocampal amnesia, and to present the associated neuropathologic findings in 1 patient who died because of an underlying malignancy.

## Methods

### Patient Identification, Serum, and CSF Samples

All 3 patients were identified by screening accompanying clinical descriptions of cases whose serum/CSF samples were sent for diagnostic testing of antineuronal antibodies between 2016 and 2019 to participating laboratories in Lübeck, Kiel (Institute of Clinical Chemistry, University Hospital Schleswig-Holstein, Kiel/Lübeck) and Vienna (Division of Neuropathology and Neurochemistry, Department of Neurology, Medical University of Vienna). AMPAR abs were identified using an in-house tissue-based assay and in-house cell-based assays (CBA) transfected with the GluA1 and GluA2 subunit of the AMPAR, as described previously.^2^ All patients were personally examined by participating neurologists in Kiel, Vienna, or Klagenfurt.

### Standard Protocol Approvals, Registrations, and Patient Consents

This study was approved by the Ethics Committee of the Medical University of Vienna (1,636/19 and 1,123/15), Lübeck (13–162), and Kiel (B337-13).

### Serum IgG Biotinylation and Competition Assay

IgG was isolated from patients' or healthy control sera with protein A/G magnetic beads (88802, Thermo Fisher Scientific) and subsequently biotinylated with a sulfo-NHS-biotinylation kit (21425, Thermo Fisher Scientific) according to manufacturer's recommendations. These biotinylated human IgG samples were then used for competition assays to detect possible recognition of the same epitopes, as previously reported.^5^ Briefly, rat brain sections were blocked and incubated with serum samples overnight at 4°C (dilution 1/5). After washing, the sections were then incubated with biotinylated human IgG (serial dilutions from 1/2 to 1/10 in 5% normal donkey serum) overnight at 4°C, and the reactivity was developed with streptavidin-horseradish peroxidase solution followed by 3,3'-diaminobenzidin.

### Neuropathology

Neuropathologic analysis was performed on formalin-fixed, paraffin-embedded tissue sections of human brain autopsy material. In total, 3–6 μm tissue sections were stained with hematoxylin and eosin, and Luxol fast blue and nuclear fast red staining. Immunohistochemistry was performed manually in a humidified chamber for complement C9 neoantigen (C9neo, polyclonal rabbit 1:2000, from Professor Paul Morgan, Cardiff, UK), GluA1 (AMPAR1, rabbit clone C3T 1:20; Merck/Millipore), GluA2/3 (AMPAR2/3, polyclonal rabbit 1:200; Merck/Millipore), Granzyme B (GranB, mouse clone GZB01 1:1,000; LabVision/Thermo Fisher Scientific), GRIK2 (kainate receptor 2, polyclonal rabbit 1:500; Sigma-Aldrich), and human Leukocyte antigen (HLA) (mouse clone HC10, 1:1,000, from Professor Hans Lassmann, Vienna, Austria), using an avidin-biotin-complex method. Enzymatic pretreatment with proteinase type 24 was used for C9neo staining; heat-induced epitope retrieval with ethylenediaminetetraacetic acid buffer pH 9 was used for GluA1, Granzyme B, and HLA-I stainings; and heat-induced epitope retrieval with citrate buffer pH 6 was used for GluA2/3 and GRIK2. Immunohistochemistry for the following primary antibodies was performed on an automated platform Autostainer Link 48 and Envision FLEX + detection kit (Dako/Agilent) and used according to manufacturer's recommendations: alpha-synuclein (mouse clone 5G4 1:4,000; Analytik Jena), beta-amyloid (mouse clone 6F/3D 1:100; Dako/Agilent), CD3 (rabbit clone SP7 1:100; NeoMarkers/Thermo Fisher Scientific), CD4 (mouse clone 4B12 1:100; Dako/Agilent), CD8 (mouse clone C8/144B 1:100; Dako/Agilent), CD20 (mouse clone L26 1:400; Dako/Agilent), CD68 (mouse clone KP1 1:5,000; Dako/Agilent), CD79a (mouse clone JCB117 1:100; Dako/Agilent), HLA-DR (mouse clone CR3/43 1:400; Dako/Agilent), IgG (polyclonal rabbit 1:16,000; Dako/Agilent), CD45/leucocyte common antigen (LCA; mouse clone 2B11+PD7/26 1:2000; Dako/Agilent), Map2 (microtubule-associated protein-2, mouse clone AP20 1:4,000; Merck/Millipore), pTDP-43 (transactive response DNA-binding protein 43, phospho-Ser409/410, mouse clone 11-9 1:20,000; Cosmo Bio), Tau (phospho-Ser202/Thr205, mouse clone AT8 1:200; Thermo Fisher Scientific), and TPPP/p25 (mouse clone 6C10 1:2,000, from Professor Gabor Kovacs, Toronto, Canada). Heat-induced epitope retrieval (HIER) with target-retrieval solution low pH (Dako/Agilent) was used for pretreatment of alpha-synuclein, CD8, CD20, CD68, CD79a, HLA-DR, IgG, LCA, TDP-43, and TPPP/p25 staining, and target-retrieval solution high pH (Dako/Agilent) was used for pretreatment of CD3, CD4, and Map2 staining. Concentrated formic acid pretreatment for 1 minute was used for alpha-synuclein and pTDP-43 staining in addition to HIER. Approximately, 80% formic acid (aqueous solution) for 1 hour was used for beta-amyloid pretreatment. Tau staining needed no pretreatment. Image acquisition was performed on a NanoZoomer 2.0-HT digital slide scanner C9600 (Hamamatsu Photonics).

### Data Availability

Anonymized data not published within this article will be made available by request from any qualified investigator.

## Results

We identified 3 patients (1 woman and 2 men; mean age at symptom onset: 44 years; range 31–69 years) with a purely amnestic syndrome and high titers of AMPAR abs demonstrated with brain immunohistochemistry and in-house cell-based assays (table). Two patients had antibodies in serum and CSF (1 and 3), and 1 patient had antibodies only in CSF (2). The clinical presentation in all 3 patients was characterized by an acute-to-subacute onset of short episodes of poor recollection, evolving into complete global amnesia reminiscent of the clinical syndrome known as transient global amnesia (median time between first symptoms and full-fledged global amnesia 7.3 days, range 1–16 days). Comprehensive cognitive and neuropsychological assessment revealed severe and isolated deficits in visual short-term memory and auditory-verbal as well as figural long-term memory and thus hippocampal dysfunction. Cognitive performance, attention, and concentration; executive and verbal function; and visuoconstructive skills were unaffected. None of the patients had epileptic seizures, change of behavior, or psychiatric symptoms. While patient 1 had no visible changes on MRI imaging, patient 2 showed uni- and patient 3 bilateral increased fluid-attenuated inversion recovery (FLAIR)/T2 signal in the hippocampus. EEG and CSF analyses were unremarkable. Two patients showed substantial neurologic improvement after immunotherapy, 1 patient had a relapse 4 months after the initial event that fully responded to immunotherapy. One patient (3) had an adenocarcinoma of the lung and only partially responded to immunotherapy. She died 3.5 months after disease onset because of tumor progression.

**Table:**
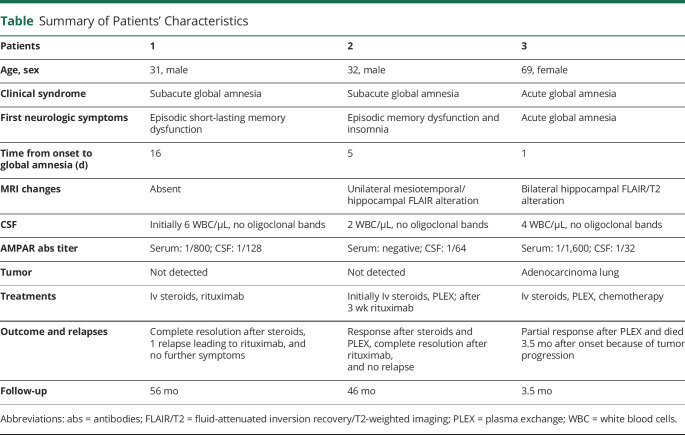
Summary of Patients' Characteristics

The antibodies of 3 patients recognized different epitopes of the AMPAR, 2 labeled cells transfected with the GluA1 subunit, and 1 recognized cells transfected with the GluA2 subunit. Immune competition experiments demonstrated that preincubation of tissue with serum of patient 3 (recognizing GluA1 subunit) did not prevent the binding of a biotinylated AMPAR serum (also recognizing the GluA1 subunit) derived from a patient with classic symptoms of AMPAR encephalitis.

**Figure 1:**
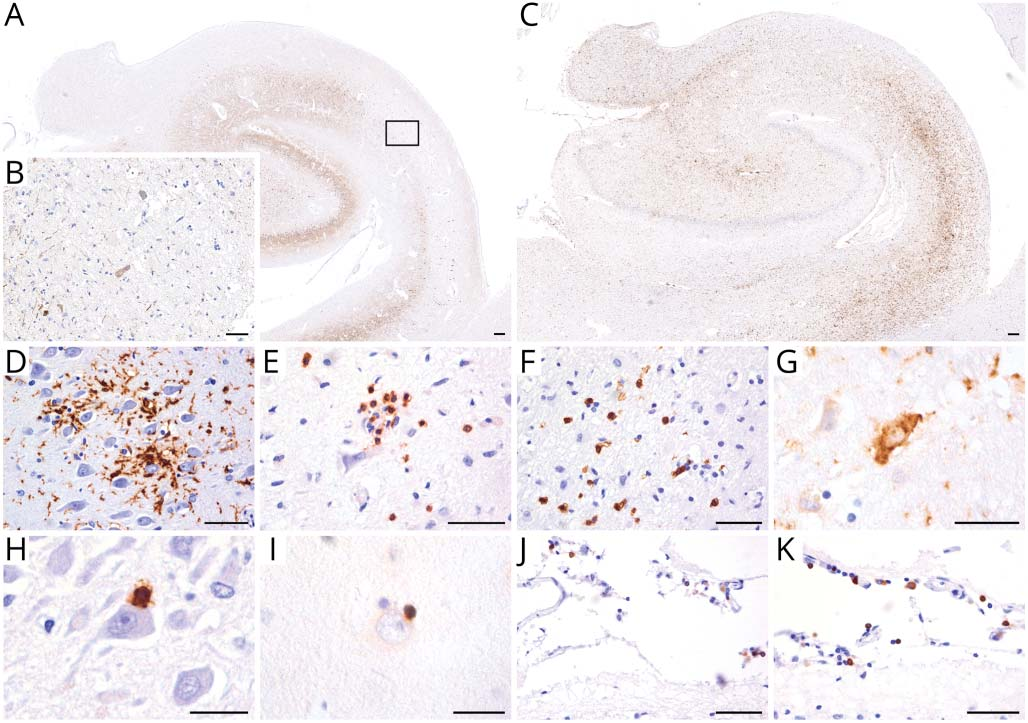
Neuropathologic Features of AMPAR Encephalitis The left hippocampus shows hippocampal sclerosis with subtotal neuronal loss in the CA1 and CA4 sectors (A, rectangle enlarged in B; neuronal marker Map2) accompanied by microglia activation (C and D, HLA-DR). Parenchymal inflammatory infiltrates in the hippocampus are composed of CD3^+^ (E) and CD8^+^ (F) T cells. Single neurons show an upregulation of HLA Class I antigen (G). Some CD8^+^ (H) and Granzyme B+ (I) cytotoxic T cells are shown in close apposition to neurons. B cells (J, CD20) and plasma cells (K, CD79a) are mainly restricted to the meninges. Scale bars in A and C: 200 μm. Scale bars in B, D–F, and J–K: 50 μm. Scale bars in G–I: 20 μm. AMPAR = alpha-amino-3-hydroxy-5-methyl-4-isoxazolepropionic acid receptor.

**Figure 2:**
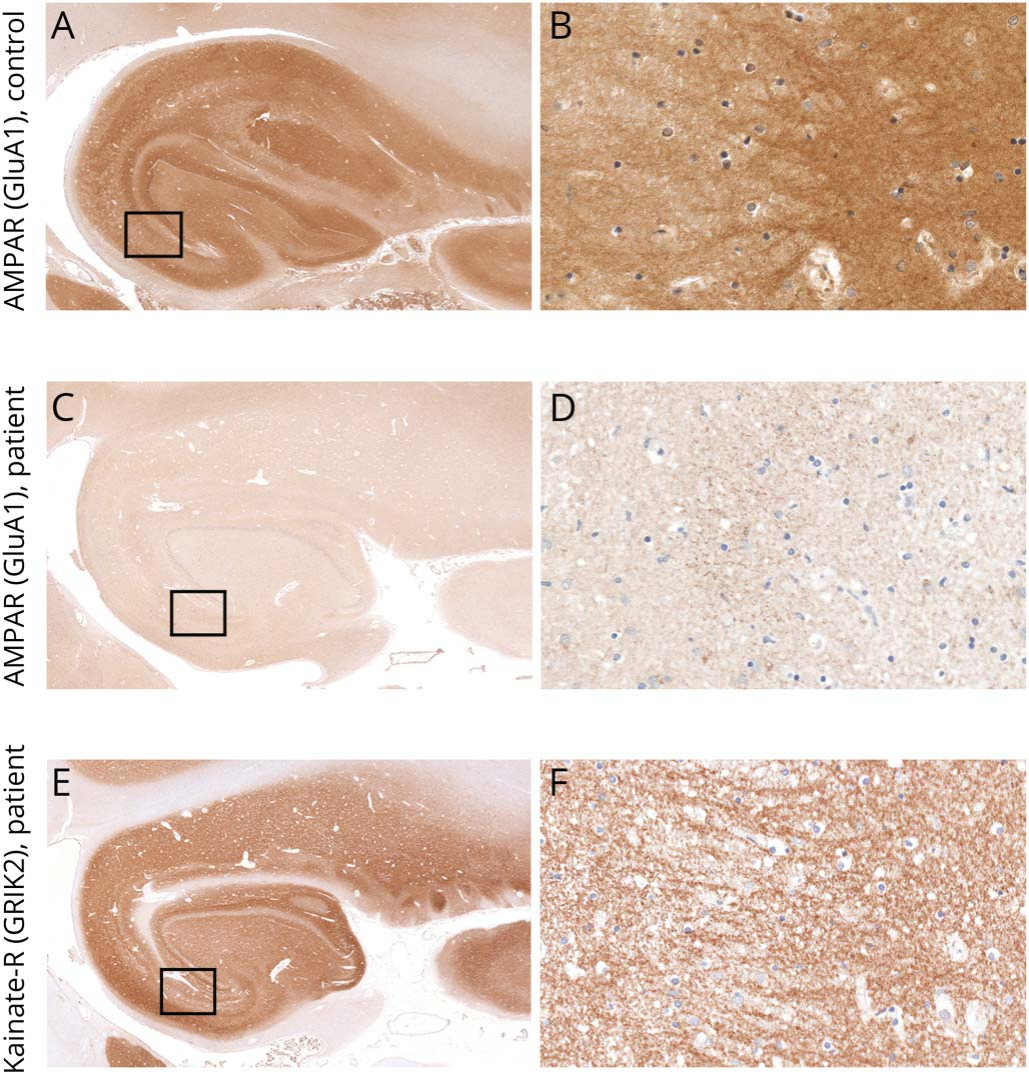
Decrease of AMPAR Density in Human Hippocampus In Vivo Compared with a control hippocampus (A, rectangle enlarged in B; GluA1), the hippocampus of the AMPAR encephalitis patient shows a significantly reduced AMPAR immunoreactivity (C, rectangle enlarged in D; GluA1), whereas the synaptic density is not altered (E, rectangle enlarged in F; GRIK2). Scale bars in A, C, and E: 1 mm. Scale bars in B, D, and F: 50 μm. AMPAR = alpha-amino-3-hydroxy-5-methyl-4-isoxazolepropionic acid receptor.

Neuropathologic analysis of the brain autopsy tissue of patient 3 revealed unilateral hippocampal sclerosis with subtotal loss of neurons in the CA1 and CA4 sectors of the left hippocampus (figure 1, A and B) accompanied by microglial activation (figure 1, C and D); astrogliosis; and moderate meningeal, perivascular, and parenchymal inflammatory infiltrates. The parenchymal inflammation was mainly composed of CD3/CD8+ T cells (figure 1, E–F). HLA Class I antigen was upregulated in single neurons (figure 1G), some of them with apposed CD8+/GranB + cytotoxic T cells (figure 1, H and I). B cells and plasma cells were mainly restricted to the meninges (figure 1, J and K). IgG deposits were detectable in preserved CA1 neurons, the subiculum, and occasionally in the isocortex. No deposits of complement C9neo were visible (data not shown). In addition, we found some perivascular inflammatory infiltrates in the basal ganglia, dentate nucleus, and brainstem. Few parenchymal infiltrates were visible in the formatio reticularis in the medulla oblongata. In addition, we found mild inflammatory infiltrates in the parietal and occipital meninges. The neurons in the prefrontal cortex and amygdala appeared well-preserved. No significant neurodegeneration-associated protein aggregates were detected; particularly, no pTDP-43, beta-amyloid, or alpha-synuclein deposits were visible. We only identified single age-related tau-positive neurofibrillary tangles, neuropil threads, and isolated perivascular and subpial astrocytes. To determine whether there was a decrease of expression of AMPAR, we stained the hippocampus sections of our patient and 7 age-matched controls (3 men and 4 women) without hippocampal pathology and 3 patients (1 man and 2 women) with hippocampal sclerosis, with commercial anti-GluA1 (AMPAR1) and anti-GluA2/3 (AMPAR2/3) antibodies. Compared with controls (figure 2, A and B), the expression of AMPAR was substantially decreased in the patient's hippocampal formation (bilateral) (figure 2, C and D), whereas the number of synapses was not altered, as demonstrated with a commercial kainate receptor antibody (figure 2, E and F).

## Discussion

We report 3 patients with AMPAR encephalitis whose clinical syndrome can be described as autoimmune global amnesia characterized by an acute-to-subacute, global amnestic syndrome without seizures, behavioral, or psychiatric abnormalities that lasted for up to 3 weeks and completely resolved after immunotherapy in the 2 cases without tumor. Acute isolated amnesia as clinical presentation of AMPAR encephalitis has previously been described in a 92-year-old woman, who remained stable after 6 cycles of IVIg.^6^ Although AMPARs are widely expressed in the brain, most patients with AMPAR encephalitis present with neurologic deficits that are restricted to the limbic system, including limbic encephalitis, seizures, behavioral, and memory deficits. Whether this is related to the density or composition of subunits of AMPARs or brain-region-dependent variations of compensatory mechanisms is unclear.^7^ AMPARs are heterotetrameric receptors composed of variable combinations of 4 subunits, GluA1-4. Our data from in-house CBAs and immunocompetition studies do not suggest that the isolated amnestic syndrome of our 3 patients is because of a specific receptor subunit and confirm the epitope diversity within the receptor that was described for AMPAR encephalitis.^8^

Amnestic syndrome refers to an impairment of memory and can be classified as anterograde or retrograde. It can be observed after trauma, bleeding, ischemia, or inflammation (viral or autoimmune, e.g., in limbic encephalitis associated with adenylate kinase 5 antibodies^9^) and may be a symptom of a psychiatric disorder (dissociative amnesia) or acute toxic metabolic disorders.^10,11^ One of the most frequent forms is transient global amnesia (TGA) that is characterized by sudden onset of short-lasting (<24 hours) anterograde amnesia in the absence of other neurologic deficits.^12-14^ An impaired venous blood flow affecting hippocampal function through congestive ischemia has been proposed as a possible pathophysiologic mechanism.^15^ Shorter lasting episodes of global amnesia over minutes to few hours can occur as epileptic syndrome (transient epileptic amnesia).^16^ In contrast to TGA, the amnestic syndrome of our patients developed over a period of 1 to 16 days and lasted for several weeks to months although at the peak of disease patients were virtually indistinguishable from patients with TGA. In 1 patient (1) in light of normal MRI and CSF without clear inflammatory changes, a diagnosis of dissociative fugue was entertained. However, the MRI showing an increased FLAIR/T2 signal in the medial temporal lobes in the other 2 patients made the final diagnosis more straightforward.

The encoding, consolidation, and retrieval of mnemonic information is critically dependent on a large bidirectional network of brain areas that includes neocortical association regions, subcortical nuclei, and the medial temporal lobe, including the hippocampus. The hippocampus is a central node in the memory network and the site of pathology in many amnestic syndromes. Many intrahippocampal subnetworks are characterized by high densities of GluA1/2 and GluA2/3 receptors.^17^ A particularly vulnerable area is the CA1 sector, which is highly sensitive to hypoxia of diverse toxic or metabolic conditions, such as hypoglycemia or abuse of psychostimulant drugs.^18^ Neuropathologic investigation of the brain in our patient revealed unilateral hippocampal sclerosis affecting the CA1 and CA4 regions of the hippocampus. The contralateral hippocampus and other areas involved in memory, such as prefrontal cortex, cingulum, or thalamus, did not show significant neuronal loss or inflammation. However, we found IgG deposition and a decrease of AMPAR immunoreactivity that was most pronounced in the hippocampal formation on both sides and not associated with a decrease of synaptic density or complement deposition. These findings are in line with previous studies that demonstrated a decrease of synaptic clusters of AMPAR subunits through antibody-mediated internalization.^1,19,20^ Whether hippocampal sclerosis in our patient is a result of the pathogenic effects of the AMPAR antibodies on synaptic function in highly vulnerable regions or secondary to a T-cell-mediated mechanism possibly triggered by a tumor-associated breach of tolerance is unclear. A hypoxic damage of the hippocampal neurons was ruled out because the patient was not affected by hypoxic-ischemic events before death.

The cases reported here have important clinical implications: AMPAR encephalitis may present with an acute/subacute global amnestic syndrome, sometimes with normal MRI that is potentially reversible with immunotherapy. The pathologic substrate of this syndrome seems to be a predominant inflammatory involvement of the CA1 and CA4 regions of the hippocampus accompanied by a decrease of expression of AMPARs.

## Glossary

AMPAR: alpha-amino-3-hydroxy-5-methyl-4-isoxazolepropionic acid receptor
abs: antibodies
CBA: cell-based assays
FLAIR: fluid-attenuated inversion recovery
HIER: heat-induced epitope retrieval
TGA: transient global amnesia
